# Learning curve and short-term clinical outcomes of a new seven-axis robot-assisted total knee arthroplasty system: a propensity score-matched retrospective cohort study

**DOI:** 10.1186/s13018-023-03899-y

**Published:** 2023-06-12

**Authors:** Xudong Duan, Yiwei Zhao, Jiewen Zhang, Ning Kong, Ruomu Cao, Huanshuai Guan, Yiyang Li, Kunzheng Wang, Pei Yang, Run Tian

**Affiliations:** Department of Bone and Joint Surgery, The Second Affiliated Hospital of Xi’an Jiaotong University, Xi’an, 710004 China

**Keywords:** Total knee arthroplasty, Learning curve, Robot-assisted system, Cumulative sum analysis

## Abstract

**Objective:**

The purpose of the present study was to determine the learning curve for a novel seven-axis robot-assisted (RA) total knee arthroplasty (TKA) system and to explore whether it could provide superior short-term clinical and radiological outcomes compared with conventional surgery.

**Methods:**

In the present retrospective study, 90 patients who underwent RA-TKA were included in robot-assisted system (RAS) group and 90 patients who underwent conventional TKA were included in the conventional group. The duration of surgery and robot-related complications were recorded to evaluate the learning curve through cumulative sum and risk-adjusted cumulative sum methods. The demographic data, preoperative clinical data, preoperative imaging data, duration of surgery, alignment of the prosthesis, lower limb force line alignment, Knee Society score, 10-cm visual analog scale pain score and range of motion were compared between the RAS and conventional groups. In addition, the proficiency group was compared with the conventional group using propensity score matching.

**Results:**

RA-TKA was associated with a learning curve of 20 cases for the duration of surgery. There was no significant difference in indicators representing the accuracy of the prosthetic installation between the learning and proficiency phases in RA-TKA group patients. A total of 49 patients in the proficiency group were matched with 49 patients from the conventional group. The number of postoperative hip–knee–ankle (HKA) angle, component femoral coronal angle (CFCA), component tibial coronal angle (CTCA), and sagittal tibial component angle (STCA) outliers in the proficiency phase was lower than that in the conventional group, while deviations of the HKA angle, CFCA, CTCA, and STCA in the proficiency phase were significantly lower than those in the conventional group (*P* < 0.05).

**Conclusion:**

In summary, from the learning curve data, 20 cases are required for a surgeon using a novel seven-axis RA-TKA system to enter the proficiency phase. In the proficiency group, compared with the conventional group using propensity score matching, the RAS was found to be superior to the conventional group in prosthesis and lower limb alignment.

## Introduction

Total knee arthroplasty (TKA) is recognized as an effective treatment for various symptomatically advanced knee diseases. It is effective in reducing joint pain and improving joint function in such patients [1]. Although conventional surgical methods and prosthetic materials have continuously improved, 20% of patients remain dissatisfied with the results of surgery, related to poor placement of the prosthesis and poor lower limb alignment following surgery [2]. Both accurate placement of the prosthesis and good lower limb alignment are key factors affecting postoperative knee function, and stability and long-term survival of the prosthesis following TKA [3]. In recent years, multiple studies have demonstrated that robot-assisted (RA) TKA systems can provide more accurate prosthesis positioning and alignment of the lower limb than conventional surgical techniques. The principle is to establish a unique 3D skeletal model through preoperative CT scans to assist the surgeon in determining the appropriate size and type of prosthesis preoperatively, and to use the robotic arm to complete preoperative planning with high precision to improve prosthesis placement and lower limb alignment [4–7]. However, robot-assisted systems (RASs) also have a number of shortcomings, such as prolonged duration of surgery, complications related to the RAS, and increased cost [8]. It has been reported that surgeons require a considerable level of training on an RAS to optimize safety and reliability [9]. A learning curve can evaluate the trend of surgical proficiency and is closely related to surgical complexity and personal experience [10–12]. Currently, it is common for cumulative sum (CUSUM) and risk-adjusted cumulative sum (RA-CUSUM) methods to be used to analyze surgical learning curves [13, 14].

In previous studies, the learning curves and duration of surgery for RA-TKA have been partially reported, but the majority relate to RASs produced in Europe and the United States [4–8]. To the best of our knowledge, no research has been published on the learning curve characteristics of Chinese RA-TKA systems. Therefore, in the present study, the use of an RA-TKA system compared with manual TKA by an experienced surgeon was reviewed. The purpose of the study was to determine the learning curve for a novel seven-axis RAS (Jianjia, Hangzhou Jianjia Robot Co., Ltd.) and to evaluate whether the system could achieve superior prosthesis positioning and lower limb alignment than conventional surgery using propensity score matching.

## Materials and methods

### Study design

Approval for this retrospective cohort study was granted by the Ethics Committee of our hospital (Permit Number: 2021–028). Patients from our hospital with unilateral TKA were enrolled from January 2021 to June 2022. By June 2022, 90 consecutive patients had undergone TKA using conventional surgical techniques, and 90 patients had received RA-TKA using a Jianjia RAS. All surgery was performed by a clinician experienced in conventional surgical techniques using the same type of knee prosthesis (Zimmer-Biomet LPS-FLEX). Patients with incomplete clinical or radiographic data were excluded.

### Surgical techniques

A three-dimensional model of the whole lower limb was established from CT data prior to TKA with the RAS, the operation guided after calculation of the osteotomy angle and osteotomy volume. An anterior median approach was adopted. Firstly, two steel needles with a diameter of 3.0 mm were driven vertically into the femur approximately 5 cm above the front femoral articular line. A guide plate was inserted to which a femoral reflection ball was connected, and the receiver position adjusted to stabilize the received signal. A positioning needle with a reflection ball was used to complete registration of spatial positioning, achieving registration between the real bone and the three-dimensional model. After registration, the operator verified the accuracy of registration, following which the robotic arm was positioned, the osteotomy guide plate connected to the robotic arm was aligned with the pre-defined line of osteotomy, and the plate inserted to complete the distal femoral osteotomy after verification that the osteotomy volume matched that calculated in preoperative planning. The position of the robotic arm was then adjusted using the guidance of the navigation system. The required anterior and posterior femoral osteotomy was completed after insertion of the respective plate using the robotic arm at the predetermined site of osteotomy. Secondly, two steel needles with a diameter of 3.0 mm were driven vertically into the tibia approximately 5 cm below the anterior tibial joint line. After registration and verification of tibial spatial positioning, correct positioning of the robotic arm was confirmed. The robotic arm was fixed and the osteotomy guide plate inserted. Osteotomy of the tibial plateau was performed after verification that the osteotomy volume matched that defined in preoperative planning. Finally, the soft tissues were balanced in both extension and flexion of the knee to achieve equal medial and lateral gaps to within ± 2 mm, respectively. The RAS was used for quantitative evaluation and verification of lower limb alignment, following comparison with the model.

### Follow-up and outcome measures

All patients underwent a CT scan 7 days after surgery to measure the following four angles to evaluate the position of the knee prosthesis: (1) Component tibial coronal angle (CTCA, neutral = 90°): the angle between the tibial component and tibial mechanical axis in the coronal plane; (2) Component femoral coronal angle (CFCA, neutral = 90°): the angle between the femoral component and femoral mechanical axis in the coronal plane; (3) Sagittal tibial component angle (STCA, neutral = 90°): the posterior angle between the tibial component and tibial mechanical axis in the sagittal plane; (4) Sagittal femoral component angle (SFCA, neutral = 90°): the angle between the femoral component and femoral mechanical axis in the sagittal plane.

Both prior to, and 12 weeks following surgery, full-length anteroposterior radiographs of both lower limbs were acquired and the hip–knee–ankle (HKA) angle (the angle from the center of the hip to the center of the knee to the center of the ankle, neutral = 180°) measured. These angles were measured independently by three trained radiologists, in random order. The mean value of the measurements was recorded. A deviation between each angle and the neutral position of ± 3° was considered acceptable. After completion of the measurements, the deviations (absolute value) between the measured values and the neutral angle values of HKA, SFCA, STCA, CTCA, and CFCA, were recorded, and the ratio of the abnormal value of each angle was calculated. In addition, the clinical results both preoperatively and 12 weeks after surgery were evaluated using the Knee Society Clinical Rating System (KSS) clinical scores, 10-cm visual analog score (VAS) for pain, and range of motion (ROM) values. Any observed complications were recorded.

### CUSUM analysis

CUSUM control charts are used to calculate the sequential difference between a data point and the cumulative mean value. The change in trend of the duration of surgery was monitored using this method, as it could not be evaluated using other methods. Duration was defined as the time from the initial surgical incision to final wound closure [15]. The cumulative sum was calculated as follows: CUSUM = ${\sum }_{i=1}^{n}({X}_{i} - U)$, where ${X}_{i}$ represented the duration of surgery for each patient, $U$ represented the mean duration for all cases, and *n* represented the sequence number of each operation. Surgery that was of greater duration increased the CUSUM value, while shorter surgery reduced the CUSUM value [16]. Where *P* < 0.05, the fitting was considered successful, with the success of fitting judged by the closeness of *R*^2^ to 1. With the inflection point of the curve representing the minimum number of surgical cases required to cross the learning curve threshold, the curve was divided into two different phases: the learning phase and proficiency phase.

### RA-CUSUM analysis

As an alternative to the CUSUM method, RA-CUSUM can be used to explain differences between the actual and predicted incidence of events [17]. In the present study, coronal and sagittal deviation angles of the prosthesis greater than 3° and deviation of the postoperative HKA angle greater than 3° were considered surgical risk factors. Where one of these defined risk factors was observed following surgery, the operation was considered a failure. Univariate analysis was used to evaluate all preoperative factors associated with the RA-TKA system. Using univariate analysis, where *P* < 0.1, multivariate logistic regression was used to calculate the probability of failure of surgery. The risk-adjusted cumulative sum was calculated as follows: RA-CUSUM = ${\sum }_{i=1}^{n}\left({X}_{i} -\uptau \right)+{(-1)}^{{X}_{i}}{P}_{i}$, where ${X}_{i}$ represented the failure of each patient, using a value of 1 for failure, and 0 for success. ${P}_{i}$ was the predicted failure rate for each patient calculated using a logistic regression model, while $\tau$ represented the overall failure rate of the surgical procedure. The operations were arranged in chronological case order from the first case to the last. A line chart was plotted with the order as abscissa and the RA-CUSUM value as ordinate. If the surgery was considered a failure, the RA-CUSUM value increased, whereas the RA-CUSUM declined for each successful operation. Finally, the RA-CUSUM analysis method was used to verify the grouping of the CUSUM analysis method.

### Group matching

To further compare the surgical techniques, patients in the proficiency RA group were matched with patients undergoing conventional surgery using propensity scores. Propensity score matching (PSM) included the following parameters: age, sex, BMI, surgical side, preoperative HKA angle, and deviation in preoperative HKA angle, VAS, ROM, and KSS. A match tolerance of 0.02 was used to set the PSM criteria.

### Statistical analysis

All data were analyzed using SPSS (v25.0 for Windows; SPSS Inc., Chicago, IL, USA). Measurement data included mean and standard deviation values, with classified variables expressed as percentages. Continuous data were analyzed using an independent sample t-test, and classified data using chi-square or Fisher tests. Differences were considered statistically significant where *P* < 0.05.

## Results

A total of 90 consecutive RA primary unilateral TKA patients, including 31 males and 59 females, were followed-up for 12 weeks. No complications, such as periprosthetic fracture, aseptic loosening, periprosthetic infection, or dislocation, were observed.

**Fig. 1:**
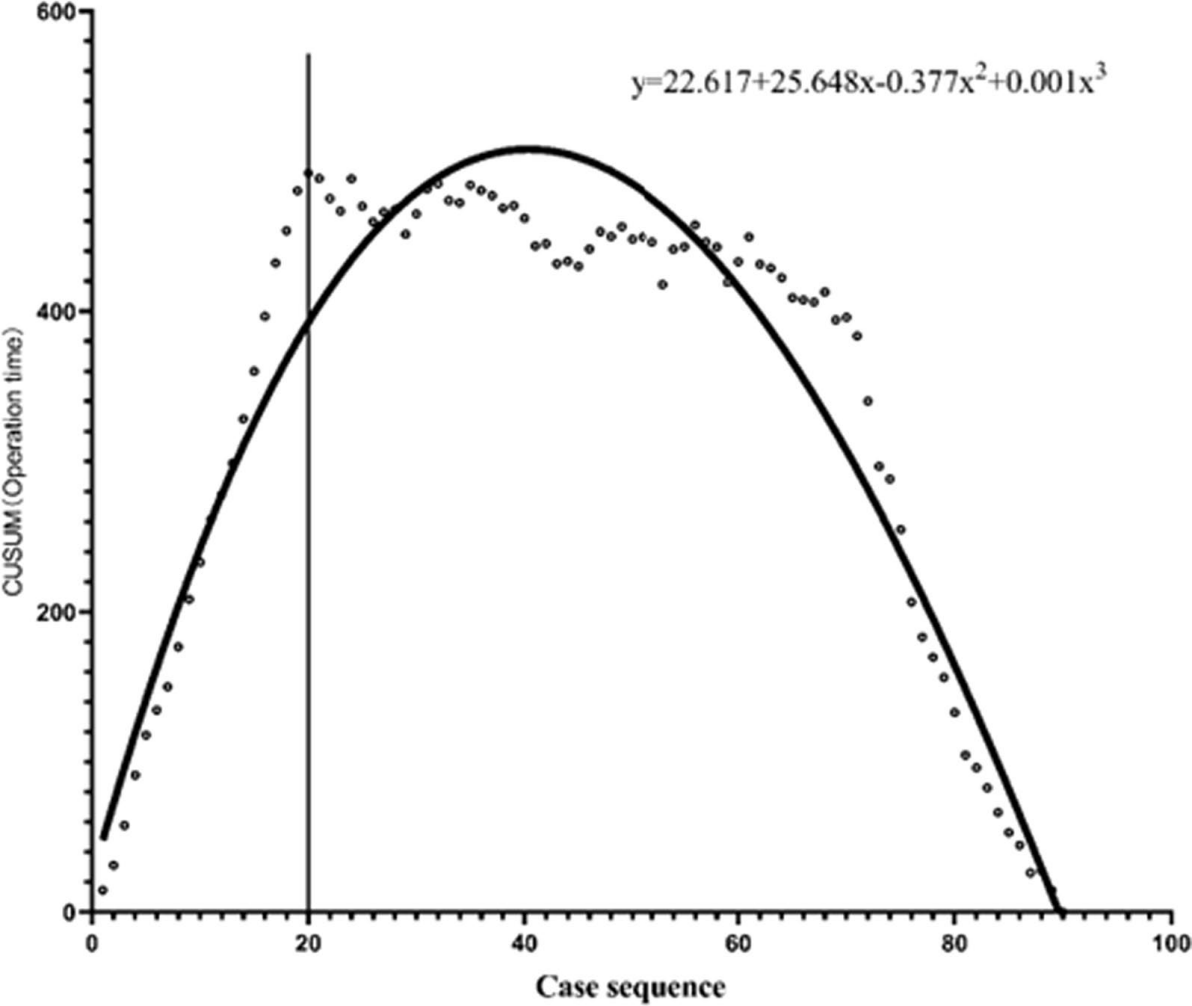
CUSUM analysis of the duration of surgery in the robot-assisted TKA system

The learning curve was analyzed using the CUSUM method. From the CUSUM chart (Fig. 1), the CUSUM peak occurred after 20 cases, allowing the learning curve to be divided into learning and proficiency groups. The CUSUM learning curve was fitted to the following cubic curve equation: CUSUM (Duration of surgery) = 22.617 + 25.648*x*−0.377*x*^2^ + 0.001*x*^3^, *R*^2^ = 0.924.

**Fig. 2:**
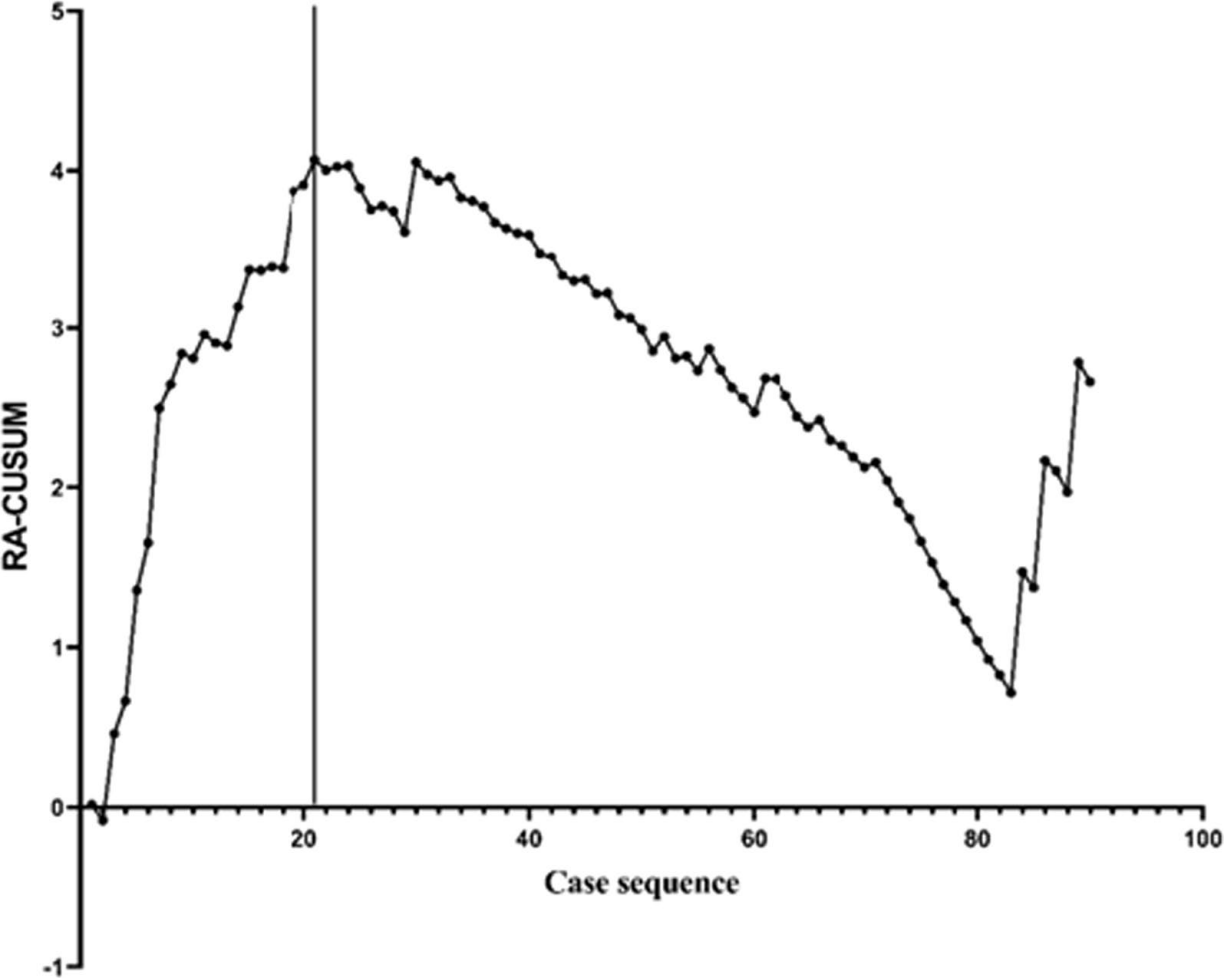
RA-CUSUM analysis of robot-assisted TKA failure

However, a decline in the CUSUM value does not represent successful surgery. Therefore, the RA-CUSUM method was used to evaluate the rate of failure of surgery. From the RA-CUSUM plot (Fig. 2), the graph reached a peak with the 21st case, representing the greatest rate of failure of the surgery, but also indicating that the rate of failure started to decline from this case onwards, representing proficiency for the RA-TKA system. Combined with the general trend and the results of the CUSUM and RA-CUSUM curves, a learning curve for the RA-TKA system was determined. The curve is divided into two groups, the first phase representing the learning phase, which spanned 20 cases (1–20), and the second phase representing the proficiency phase (cases 21–90) which began immediately after.

The two phases were compared in terms of demography, preoperative clinical results, preoperative radiographic data, duration of surgery, postoperative clinical results, and postoperative radiographic data (Table 1). There was no significant difference in age, surgical side, body mass index (BMI), sex, preoperative HKA angle, deviation in preoperative HKA, preoperative VAS, preoperative ROM, or preoperative KSS between the two groups (*P* > 0.05). The duration of surgery in the learning group (128.00 ± 7.50 min) was significantly longer than that in the proficiency group (96.37 ± 14.63 min) (*P* < 0.001). In terms of clinical results, there was no significant difference in the postoperative ROM, KSS, or VAS scores between the two groups. In terms of radiographic results, there was no significant difference in postoperative HKA angle or deviation in HKA angle, or deviation in CTCA, CFCA, STCA, or SFCA between the two groups.

**Table 1:** Comparison of the clinical and radiographic data between the two groups using the RA-TKA system

	Learning group	Proficiency group	Statistic	*P* value
N	20	70		
Age	70.50 ± 5.54	67.80 ± 7.76	*t* = 1.754	0.087
BMI (kg/m^2^)	27.56 ± 2.53	26.28 ± 3.69	*t* = 1.784	0.081
Male (%)	35.0	34.3	χ^2^ = 0.004	0.953
Surgical side				
Left (%)	45.0	52.9		
Right (%)	55.0	47.1	χ^2^ = 0.384	0.535
Preoperative HKA angle	170.22 ± 6.18	172.52 ± 5.68	*t* = − 1.568	0.121
Preoperative HKA angle deviation	10.39 ± 5.24	8.33 ± 4.32	*t* = 1.787	0.077
Preoperative VAS	6.03 ± 1.55	6.82 ± 1.82	*t* = − 1.769	0.080
Preoperative ROM	109.20 ± 13.99	101.16 ± 19.57	*t* = 1.714	0.090
Preoperative KSS	45.30 ± 17.78	47.04 ± 16.83	*t* = − 0.403	0.688
Operation time	128.00 ± 7.50	96.37 ± 14.63	*t* = 13.056	0.000*
Postoperative HKA angle deviation	2.24 ± 0.78	1.83 ± 0.93	*t* = 1.956	0.058
Postoperative CTCA deviation	1.31 ± 0.99	1.28 ± 0.89	*t* = 0.102	0.919
Postoperative CFCA deviation	1.24 ± 0.87	1.04 ± 0.84	*t* = 0.899	0.371
Postoperative STCA deviation	2.14 ± 1.78	1.69 ± 1.20	*t* = 1.329	0.187
Postoperative SFCA deviation	2.23 ± 1.65	1.79 ± 1.46	*t* = 1.165	0.247
Postoperative ROM	119.25 ± 7.66	116.70 ± 11.75	*t* = 1.152	0.255
Postoperative KSS	60.80 ± 8.06	62.74 ± 8.63	*t* = − 0.901	0.370
Postoperative VAS	2.68 ± 1.79	2.65 ± 1.62	*t* = 0.053	0.958

*BMI* body mass index; *ROM* range of motion; *KSS* Knee Society score; *VAS* 10-cm visual analog scale

*P < 0.05

The rate of outliers of the above five angles (Table 2) was further analyzed. There was no significant difference between the two groups (*P* > 0.05).

**Table 2:** Outliers in component positions and lower limb alignment in patients operated using an RA-TKA system

Group	Percentage of knees with implant aligned outside ± 3° from neutral angle				
	STCA	SFCA	CTCA	CFCA	HKA
Learning group	10.0%	10.0%	5.0%	5.0%	5.0%
Proficiency group	5.7%	4.3%	0.0%	1.4%	2.9%
χ^2^	0.459	0.968	3.539	0.913	0.222
*P* value	0.498	0.325	0.060	0.339	0.638

In terms of complications, there was one case of poor wound healing and two cases of wound exudation in the learning group. In the proficiency group, there were three cases of poor wound healing and four cases of wound exudation. There was one case of lower limb deep vein thrombosis in the learning group and three cases in the proficiency group, which was resolved using drug thrombolysis. No RAS-related complications, such as needle infection or peri-needle fracture, were experienced in either of the two groups. There was no significant difference in the total incidence of complications between the two groups (*P* > 0.05).

The demographics, duration of surgery, and the clinical and radiographic results of RA-TKA (cases 21–90) performed by the same surgeon in the proficiency phase were compared with those of 90 consecutive cases of conventional TKA in the same period. A total of 98 patients were matched by PSM and included for analysis: (1) 49 patients in the proficiency group, and (2) 49 patients in the conventional group. Prematched and postmatched data are displayed in Tables 3 and 4. As shown in Table 4, there was no significant difference in demographic, preoperative clinical and radiographic results between the two groups (*P* > 0.05). The postoperative HKA angle deviation was 1.75 ± 0.97° in the proficiency group and 3.07 ± 2.43° in the conventional group, a statistically significant difference (*P* < 0.001). The deviation in postoperative CFCA in the proficiency and conventional groups was 0.97 ± 0.83° and 1.81 ± 1.76°, respectively, a statistically significant difference (*P* = 0.003). There were significant differences between the proficiency group and the conventional group for deviation in postoperative CTCA (1.24 ± 0.86° vs. 1.70 ± 1.29°, *P* = 0.039) and postoperative STCA (1.83 ± 1.22° vs. 2.41 ± 1.43°,* P* = 0.032), but no significant difference between the two groups in the deviation in postoperative SFCA (1.87 ± 1.67° vs. 2.18 ± 2.05°, *P* > 0.05).

**Table 3:** Comparison of demographics in the unmatched data

	Proficiency group	Conventional group	Statistic	*P* value
N	70	90		
Age	67.80 ± 7.76	70.30 ± 7.49	*t* = − 2.073	0.040*
BMI (kg/m^2^)	26.28 ± 3.69	26.46 ± 3.32	*t* = − 0.320	0.750
Male (%)	34.3	23.3	χ^2^ = 2.337	0.126
Surgical side				
Left (%)	52.9	51.1		
Right (%)	47.1	48.9	χ^2^ = 0.048	0.826
Preoperative HKA angle	172.52 ± 5.68	174.87 ± 8.53	*t* = − 2.088	0.038*
Preoperative HKA angle deviation	8.33 ± 4.32	8.77 ± 4.64	*t* = − 0.616	0.539
Preoperative VAS	6.82 ± 1.82	6.51 ± 1.75	*t* = 1.097	0.274
Preoperative ROM	101.16 ± 19.57	106.71 ± 14.41	*t* = − 2.067	0.040*
Preoperative KSS	47.04 ± 16.83	43.96 ± 12.82	*t* = 1.274	0.205

*BMI* body mass index; *ROM* range of motion; *KSS* Knee Society score; *VAS* 10-cm visual analog scale

*P < 0.05

**Table 4:** Comparison of the clinical and radiographic data between the matched groups

	Proficiency group	Conventional group	Statistic	*P* value
N	49	49		
Age	69.20 ± 6.82	69.43 ± 6.94	t = − 0.161	0.872
BMI (kg/m^2^)	26.84 ± 3.77	26.44 ± 3.45	t = 0.556	0.579
Male (%)	36.7	30.6	χ^2^= 0.411	0.521
Surgical side				
Left (%)	51.0	46.9		
Right (%)	49.0	53.1	χ^2^ = 0.163	0.686
Preoperative HKA angle	172.42 ± 5.81	172.26 ± 6.65	*t* = 0.128	0.899
Preoperative HKA angle deviation	8.49 ± 4.33	8.74 ± 5.23	*t* = − 0.276	0.790
Preoperative VAS	6.75 ± 1.94	6.53 ± 1.83	*t* = 0.588	0.588
Preoperative ROM	103.86 ± 15.73	104.02 ± 15.38	*t* = − 0.052	0.959
Preoperative KSS	42.86 ± 15.50	48.12 ± 13.05	*t* = − 1.819	0.072
Duration of surgery	98.22 ± 14.42	90.33 ± 11.15	*t* = 3.032	0.003*
Postoperative HKA angle deviation	1.75 ± 0.97	3.07 ± 2.43	*t* = − 3.524	0.000*
Postoperative CTCA deviation	1.24 ± 0.86	1.70 ± 1.29	*t* = − 2.095	0.039*
Postoperative CFCA deviation	0.97 ± 0.83	1.81 ± 1.76	*t* = − 3.034	0.003*
Postoperative STCA deviation	1.83 ± 1.22	2.41 ± 1.43	*t* = − 2.171	0.032*
Postoperative SFCA deviation	1.87 ± 1.67	2.18 ± 2.05	*t* = − 0.806	0.422
Postoperative ROM	116.73 ± 11.35	116.16 ± 12.82	*t* = 0.234	0.816
Postoperative KSS	62.55 ± 7.72	61.57 ± 11.17	*t* = 0.505	0.615
Postoperative VAS	2.63 ± 1.62	3.21 ± 2.10	*t* = − 1.536	0.128

*BMI* body mass index; *ROM* range of motion; KSS Knee Society score; *VAS *10-cm visual analog scale

*P < 0.05

As displayed in Table 5, there was no significant difference in the rate of outliers of the SFCA between the two groups (*P* > 0.05). The rate of the postoperative HKA angle outliers in the proficiency and conventional groups were 4.1% and 36.7%, respectively, a difference that was statistically significant (*P* < 0.001). The rate of the postoperative CFCA outliers in the proficiency and conventional groups was 2.0% and 14.3%, respectively, a difference that was statistically significant (*P* = 0.027). The rate of postoperative CTCA outliers in the proficiency and conventional groups was 0.0% and 8.2% respectively, a difference that was statistically significant (*P* = 0.041). The rate of postoperative STCA outliers in the proficiency and conventional groups was 4.1% and 16.3%, respectively, a difference that was also statistically significant (*P* = 0.045). Additionally, the duration of surgery for the proficiency group was 98.22 ± 14.42 min, which was longer than that of the conventional group (90.33 ± 11.15 min), a difference that was statistically significant (*P* = 0.003).

**Table 5:** Comparison of outliers in component positions and lower limb alignment between the proficiency and conventional groups

Group	Percentage of knees with implant aligned > ± 3° from neutral angle				
	STCA	SFCA	CTCA	CFCA	HKA
Proficiency group	4.1%	4.1%	0.0%	2.0%	4.1%
Conventional group	16.3%	14.3%	8.2%	14.3%	36.7%
χ^2^	4.009	3.059	4.170	4.900	16.082
*P* value	0.045*	0.080	0.041*	0.027*	0.000*

*P < 0.05

## Discussion

Robotic-assisted knee replacement is the hotspot in the field of total knee replacement. Theoretically, robotic-assisted knee replacement can result in better lower limb alignment. But at the same time, it also prolongs the operation time, adds additional tests and costs, and there may be complications related to the robot-assisted system, so whether patients can benefit in the long term is controversial [18, 19]. It has been reported that surgeons require a considerable level of training on an RAS to optimize safety and reliability [9]. In a recent study, it was shown that the robotic-assisted TKAs remained cost-effective when annual revision rates < 1.6% and quality of life values were > 0.85 [20]. During the follow-up, No RAS-related complications, such as needle infection or peri-needle fracture, were experienced in either of the two groups. Therefore, in this study, we focused on the efficacy before and after the learning curve of the RA-TKA.

In the present study, surgery performed by RA-TKA was divided into learning and proficiency groups, by combining the results of analysis by CUSUM and RA-CUSUM. It is important to note that, although the RA-CUSUM curve indicates continued surgical failure after 21 patients, this is related to the greater requirement for the surgical technique at a later phase of the learning curve. There were no significant differences in demographics, preoperative clinical data, preoperative radiographic data, postoperative clinical results, or postoperative radiographic results between the two groups (*P* > 0.05). No RAS-related complications were identified in either of the learning or proficiency groups during the follow-up period, with no significant differences in the total incidence of other complications between the two groups (*P* > 0.05). This may be related to the surgeon's rich experience and the short follow-up period. The duration of surgery in the learning group was longer than that in the proficiency group, a difference that was statistically significant (*P* < 0.001).

In addition, we also compared the duration of surgery, and the radiographic and clinical results for RA-TKA in the proficiency phase and conventional group after propensity score matching. The rate of outliers for postoperative HKA angle, CFCA, STCA, and CTCA in the proficiency group was superior to that in the conventional group. Deviation of the HKA angle, CFCA, STCA, and CTCA in the proficiency group was smaller than that in the conventional group, although the duration of surgery was longer, a difference that was statistically significant (*P* < 0.05). However, there was no significant difference in VAS or KSS scores, or ROM between the two groups (*P* > 0.05). The results indicated that the RAS was more advantageous for prosthesis alignment and restoring the lower limb force lines. The results of research by Khlopas indicate that RA knee arthroplasty systems are able to achieve greater accuracy of prosthesis placement and have a shorter learning curve [21]. A comparison of an RA-TKA system with conventional TKA by Hampp et al*.* [7] verified that robotic assistance provides prosthetic alignment superior to that of conventional TKA. The present study also indicated similar conclusions. A large number of other previous studies also support this view [22–24].

A number of previous studies have evaluated the learning curve of RA-TKA systems [11, 25, 26]. However, the majority have compared the surgical results by simply dividing the sequence of the operations. This division and method of comparison has a number of limitations. In the present study, the learning curve for RA-TKA was determined by CUSUM and RA-CUSUM analyses. Using this analysis method, not only are changes in the duration of surgery considered, but also the failure rate of surgery using RA-TKA, including recovery of lower limb alignment and precision, position of the prosthesis, and duration of surgery. This is important because TKA requires accurate prosthesis alignment, and restoration of lower limb force lines to enhance patient satisfaction, reduce or prevent postoperative pain, prosthesis loosening, and increase the rate of prosthesis survival. Poor alignment can cause patients to require early revision [27, 28]. Although the learning curves of RA-TKA systems such as Mako and ROBODOC have been reported in the literature, only a few RASs registered by the National Medical Products Administration of China have been reported. Recently, a novel seven-axis RA-TKA system has been designed and developed, but no data for its learning curve have been published. Therefore, the present retrospective study was conducted to analyze its learning curve and effectiveness.

In a previous study, Naziri et al*.* [29] demonstrated that there was a learning curve to successfully operating an RA-TKA system by comparing the results of the first 20 patients and the last 20 patients using a test system. Recently, Vermue et al*.* [15] used CUSUM analysis to evaluate the learning curve of an RA-TKA system, finding that for a high-volume surgeon, the learning curve had an inflection point at 22 cases, similar to the present study. CUSUM and RA-CUSUM analysis methods are now considered reliable methods for the evaluation of surgical learning curves [16, 17, 30]. At present, no published studies have combined these two methods to determine the learning curve of RA-TKA systems. The CUSUM analysis method provides a continuous curve and a clear turning point, while the RA-CUSUM method allows additional evaluation of the change in surgical failure rate. Therefore, we used both the CUSUM and RA-CUSUM methods to determine the learning curve for an RA-TKA system for a single surgeon.

Although there is a learning curve for operating a RAS, we observed no significant difference in prosthesis alignment or the restoration of lower limb force lines before achieving the learning curve compared with after, which indicates that the system can maintain its accuracy during the process of operator training, reflecting the advantages of the system. A study published by Marchand et al*.* [31] demonstrated that an RA-TKA system improved postoperative pain and knee joint function compared with conventional methods. Li et al*.* [32] found no significant difference in WOMAC, HSS, SF-36, or KSS scores in patients that were operated using robot assistance compared with conventional surgery. In the present study, no significant difference was observed in the KSS or VAS scores, or ROM in the RA group compared with conventional surgery (*P* > 0.05). Evidence of clinical improvement due to enhanced prosthesis alignment and lower limb force line restoration due to utilization of an RA-TKA system are unlikely to be observed over only a short period of time. Secondly, the operators in the present study had rich experience with the ability to achieve highly accurate results using traditional methods. Finally, whether an improvement in radiographic results will achieve longer prosthesis survival requires confirmation.

The present study had a number of limitations. Firstly, the retrospective study design has resulted in multiple instances of research bias, including selection bias, evaluation bias, and measurement bias, which are difficult to offset. Secondly, the sample size of the study is small, with relatively few observation indicators. In the future, studies with larger numbers of samples and a larger range of indicators are required to verify the efficacy and learning curve of RASs. Finally, as a single-center study, the learning curve obtained in the present study may not be applicable to other centers.

## Conclusions

In the present study, 20 cases were required for an experienced surgeon to achieve the threshold of the learning curve of a novel seven-axis robotic-assisted TKA system for passing into a proficiency phase. Utilization of the RAS in the proficiency phase, compared with the conventional group with propensity score matching, is more advantageous than conventional surgical methods regarding lower limb force line and prosthesis alignment. Whether RASs will provide superior clinical function and radiographic performance over the long term requires further follow-up and research.

## Data Availability

The datasets generated during or analyzed during the current study are available from the corresponding author on reasonable request.

## Abbreviations

RA-TKA: Robot-assisted total knee arthroplasty
CUSUM: Cumulative sum
KSS: Knee Society score
BMI: Body mass index
RA-CUSUM: Risk-adjusted cumulative sum
ROM: Range of motion
VAS: 10-Cm visual analog scale
